# Risk of stress cardiomyopathy associated with selective serotonin reuptake inhibitors and serotonin and norepinephrine reuptake inhibitors: a real-world pharmacovigilance analysis

**DOI:** 10.1038/s41598-024-66155-1

**Published:** 2024-07-02

**Authors:** Boyu Tan, Li Chen, Sulan Yan, Huijie Pan, Jingxian Zhang, Hongyan Wei

**Affiliations:** 1 Department of Pharmacy, Shanghai Children’s Hospital, Children’s Hospital Affiliated to Shanghai Jiao Tong University School of Medicine, Shanghai, People’s Republic of China; 2 Department of Pharmacy, Guanghua Hospital Affiliated to Shanghai University of Traditional Chinese Medicine, Shanghai, People’s Republic of China; 3 Department of Pharmacy, Hunan Provincial People’s Hospital, The First Affiliated Hospital of Hunan Normal University, Changsha, Hunan People’s Republic of China; 4 Department of Cardiology, Hunan Provincial People’s Hospital, The First Affiliated Hospital of Hunan Normal University, Changsha, Hunan People’s Republic of China; 5 Institute of I Arthritis Research in Integrative Medicine, Shanghai Academy of Traditional Chinese Medicine, Shanghai, People’s Republic of China

**Keywords:** SSRIs, SNRIs, Antidepressants, Cardiomyopathy, FAERS, Pharmacovigilance, Spontaneous reporting systems, Psychology, Cardiology, Risk factors

## Abstract

Selective serotonin reuptake inhibitors (SSRIs) and serotonin and norepinephrine reuptake inhibitors (SNRIs) are reported to cause stress cardiomyopathy (SC). This study evaluated the association between SSRI/SNRI use and the occurrence of cardiomyopathy in the publicly available U.S. Food and Drug Administration Adverse Event Reporting System (FAERS) database. Disproportionate analysis and likelihood ratio tests were used to identify risk associated with SSRIs or SNRIs and the incidence of SC, using data from between from 2012 to 2022 acquired from the FAERS database. The study identified 132 individual case safety reports (ICSRs) of SC associated with SSRIs or SNRIs. Venlafaxine (48%) and fluoxetine (27%) were the most common antidepressants of the ICSRs. Approximately 80% of SC cases were reported in females, with individuals aged 45–65 years identified as a high-risk population. Both venlafaxine (ratio-scale information component [RSIC] 2.54, 95% CI 2.06–3.04) and fluoxetine (RSIC 3.20, 95% CI 2.31–4.47) were associated with SC, with likelihood ratio estimates of 3.55 (p = 0.02) for venlafaxine and 4.82 (p = 0.008) for fluoxetine. The median time to cardiomyopathy onset was 20 days, with hospitalization reported in 48.33% of patients. Venlafaxine and fluoxetine were associated with SC risk, particularly in middle-aged women. Caution should be exercised when using SSRIs or SNRIs combined with other serotonergic medications.

## Introduction

Stress-induced cardiomyopathy, also known as Takotsubo syndrome or broken heart syndrome, is an acute reversible heart failure syndrome characterized by transient regional abnormalities in the left ventricular contraction. Changes in cardiac morphology during the disease often resemble Japanese octopus traps. Therefore, it was named Takotsubo cardiomyopathy^1^. The pathophysiological mechanisms underlying of Takotsubo syndrome have not been fully elucidated. The catecholamine theory is one of the most widely accepted hypotheses. Acute stressors increase the concentrations of neuropeptides and catecholamines (dopamine, epinephrine, and norepinephrine) during the acute phase of the disease, contributing to left ventricular dysfunction^2^. Physical or emotional stress is commonly recognized as the primary cause of stress cardiomyopathy (SC). However, etiologies related to medication use have also been suggested.

The use of serotonin and norepinephrine reuptake inhibitors (SNRIs) and selective serotonin reuptake inhibitors (SSRIs) in therapeutic dosages or overdoses may cause SC^3,4^, particularly in the context of the COVID-19 pandemic. SNRIs such as venlafaxine and duloxetine elevate norepinephrine levels in neuronal tissues by blocking reuptake, exposing vulnerable patients to a surge in catecholamine levels, thereby promoting the development of SC^5^. SSRIs, including fluoxetine and sertraline, cause SC and may interact with serotonin and other neurotransmitter systems^6^. Theoretically, SNRIs seem to have a greater likelihood of inducing SC than SSRIs, because SNRIs inhibit the reuptake of both serotonin and norepinephrine. However, clinical studies directly comparing the risk of SC between these classes of antidepressants are lacking. Moreover, safety data for SSRIs and SNRIs are crucial in clinical practice, especially for patients with concurrent depression and cardiovascular diseases.

Real-world pharmacovigilance studies examining the risk of cardiomyopathy associated with different antidepressants provide valuable insights for guiding clinical drug selection. These studies bridge existing knowledge gaps and improve understanding of the safety profiles of SSRIs and SNRIs in SC. By analyzing data on a greater scale and for extended periods, these studies could clarify the potential risks and benefits associated with these medications. This knowledge will enable healthcare professionals to make better-informed choices when prescribing antidepressants to patients with cardiovascular disease.

The Food and Drug Administration Adverse Event Reporting System (FAERS) is a database maintained by the U.S. Food and Drug Administration (FDA) and is one of the largest spontaneous report databases for adverse events worldwide. FAERS collects voluntarily submitted reports on adverse events, medication errors, and product quality issues from healthcare professionals, drug manufacturers, consumers, and other individuals. Using the FAERS database, researchers, regulatory bodies, and healthcare professionals can analyze and evaluate the real-world clinical safety of medications. This database is critical in pharmacovigilance, providing valuable insights into the drug safety profiles.

This study using the FAERS database to analyze the relationship between SSRI or SNRI antidepressant medications and adverse cardiomyopathy events. By harnessing the extensive data available in the FAERS, we examined the potential associations between using these medications and cardiomyopathy. This analysis will contribute to understanding the safety profiles of SSRIs and SNRIs related to cardiomyopathy using real-world evidence derived from diverse patient populations.

## Methods

### Data source and preparation

Relevant data from the first quarter of 2012 to the fourth quarter of 2022 were downloaded from the FAERS website and imported into a local relational database. Relevant demographic, exposure, treatment, and outcome variables were extracted; duplicate data were removed from the raw data following the FDA recommendations. The cases listed in the deleted files were excluded. The FAERS data are publicly available, anonymized, and exempt from ethical committee review.

The adverse event onset time was calculated as the duration between event onset (EVENT_DT) and the initiation of drug administration (START_DT). Cases were excluded if the EVENT_DT or START_DT fields lacked year or month data, the event occurrence date was earlier than the drug start date, or adverse events occurred < 3 times.

### Identification of outcome and indications

In FAERS, the adverse events are coded using the preferred terms from the International Council for Harmonisation of Technical Requirements for Pharmaceuticals for Human Use’s international Medical Dictionary for Regulatory Activities (MedDRA)^7^. This study used narrow-scope standardized MedDRA queries (SMQs) for depression to identify drugs intended for antidepressant treatment, excluding suicide and self-injury. Adverse event reports using narrow-scope SMQ for cardiomyopathy (20000150) were retrieved (Table S1). The SMQ comprises MedDRA terms for specific medical conditions or areas of concern, including signs, symptoms, diagnoses, syndromes, and physical examination findings, such as laboratory and other physiological examination data, typically at the preferred term level. The status of the terms had no restrictions.

### Identification of exposure

The SSRIs in this study included citalopram, escitalopram, fluvoxamine, fluoxetine, sertraline and paroxetine; the SNRIs included venlafaxine, milnacipran, duloxetine, levomilnacipran, and desvenlafaxine. Because drug names in the FAERS database are reported in different forms, including trade names, generic names, and synonyms, different names of the same drug were standardized to generic names using DrugBank (version 5.1.9)^8^. A fuzzy matching algorithm was used to enhance case identification and minimize drug misclassification^9^. The cases were reviewed individually by two clinical pharmacists.

In the FAERS database, the codes used to describe a drug's reported role in adverse events include primary suspect drug, secondary suspect drug, concomitant, and interacting drug. This study considered only cases in which a drug was identified as a primary or secondary suspected drug.

### Statistical analysis

Data management, statistical analysis, and visualization were performed using R version 4.0.2^10^. The pharmsignal and pvLRT software packages were used for pharmacovigilance analysis. The DrugBank (version 5.1.9) database was used to determine the severity of drug–drug interactions. Descriptive analysis was used to summarize the demographic characteristics of the individual case safety reports (ICSRs). The Bayesian confidence propagation neural network information component (BCPNN) statistic was utilized and estimated using Markov Chain Monte Carlo simulation^9^. This method calculates the exponential information component, the ratio-scale information component (RSIC), to report disproportionality estimates. This approach applies to small sample sizes. In this study, potential positive signals needed to have at least three reports for each drug–event pair, with an RSIC estimate > 2 and a 95% confidence interval (CI) lower bound > 1 (α = 0.05).

A likelihood ratio test (LRT) for further signal detection was used to validate the robustness of the results obtained using the BCPNN method. The LRT method assumed that the report count of drug–adverse event combinations follows a Poisson distribution. The LRT method was used to (i) identify adverse events with greater reporting rates than other adverse events associated with a specific drug and (ii) identify drugs with greater reporting rates of a specific adverse event than other drugs. This approach has been shown to control family-wise type-I errors and exhibit good performance characteristics for power and sensitivity^11^.

### Ethics statement

The data included in this study were obtained from the FAERS database, which is publicly available and anonymous, and therefore the study was exempt from approval by the institutional ethics committee.

## Results

### Descriptive analysis

**Figure 1:**
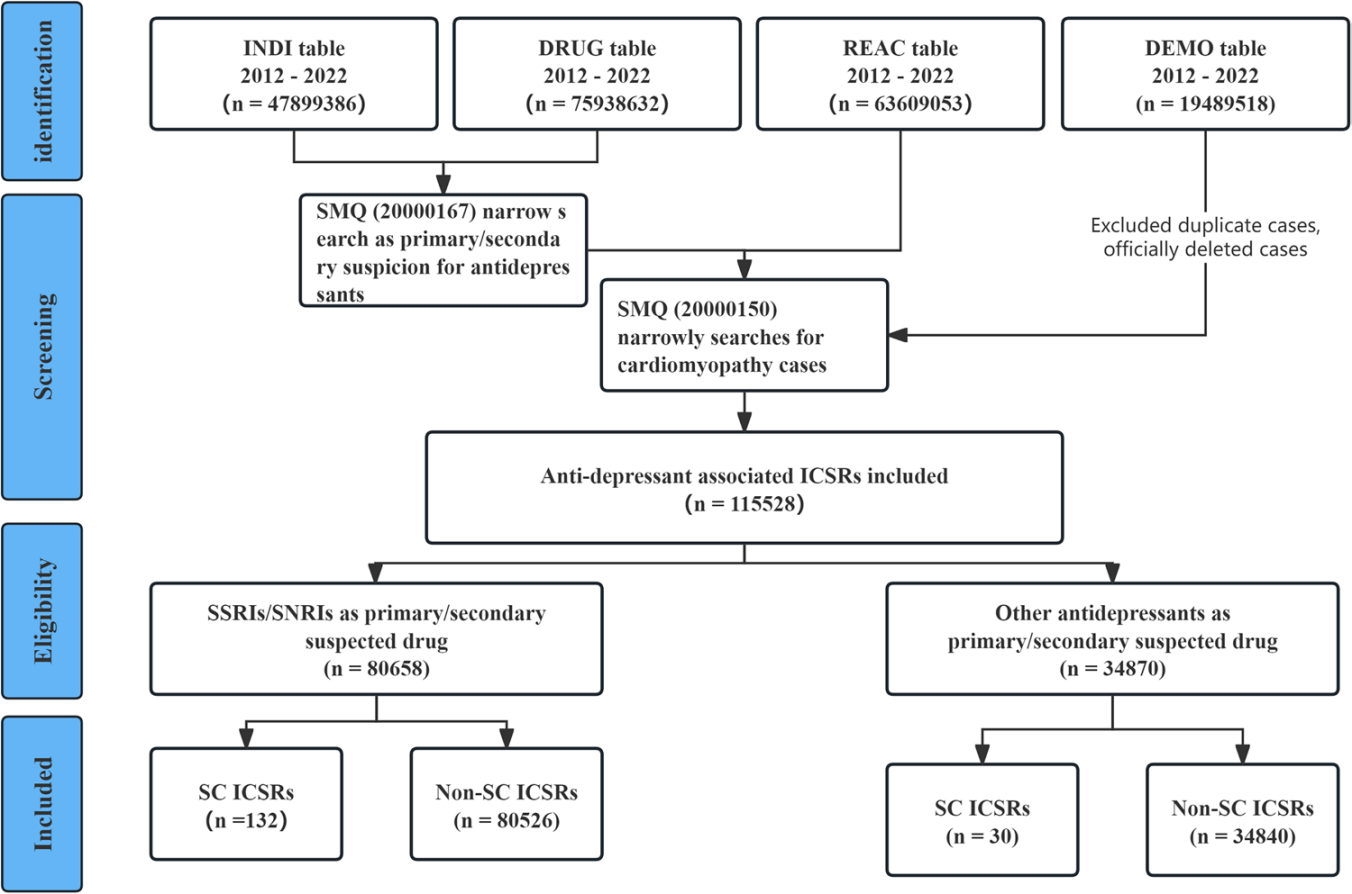
Flowchart for the identification of ICSRs of stress cardiomyopathy associated with SSRIs/SNRIs in the FAERS database.

Between January 2012 and December 2022, 115528 ICSRs involving antidepressants were recorded in the FAERS database. After cleaning data and removing duplicates, 132 ICSRs showed that using SSRIs and SNRIs was associated with SC (Fig. 1).

The demographic characteristics related to suspected drugs, age, gender, reporter occupation, and geographic region of reporting are summarized in Table 1. Six antidepressants were implicated in 132 ICSRs involving the SC. Venlafaxine (48%), fluoxetine (27%), and paroxetine (13%) were the top three suspected antidepressant medicines of the ICSRs. A review of these timelines found that 2016 (n = 32, 24%) and 2022 (n = 27, 20%) had more reports. Of 132 ICSRs, 59 instances (45%) were reported to exhibit a positive dechallenge response. However, the ADRs associated with the rechallenge response remain unclear. In general, the age distribution of ICSRs was concentrated primarily in the 45–65 age group, accounting for approximately 41% of the total (54 cases of SC reported). In addition, 24 instances (18%) occurred in the 18–45 age group, 16 (12%) in the 65–75 age group, 12 (9.1%) in the ≥ 75-year age group, 3 (2.3%) in the < 18 age group, and 23 (17%) in which the age was unknown. Female patients accounted for 105 (80%) of all SC cases; males accounted for only 6 instances (4.5%). The remaining 21 instances were cases in which gender information was not recorded. Adverse events were registered primarily by healthcare professionals and originated predominantly in Europe and North America. Detailed baseline characteristic stratifications (age group, gender, and region) for SSRIs and SNRIs are show in Table S1.

**Table 1:** The demographic characteristics of SC events associated with SSRIs and SNRIs.

Characteristics	SC (n = 132)	Non-SC (n = 80,526)
SSRIs		
Fluoxetine	35 (27%)	8,085 (10%)
Paroxetine	17 (13%)	6,244 (7.8%)
Sertraline	4 (3.0%)	16,950 (21%)
SNRIs		
Venlafaxine	63 (48%)	17,401 (22%)
Duloxetine	10 (7.6%)	14,562 (18%)
Milnacipran	3 (2.3%)	535 (0.7%)
Year of report		
2012	2 (1.5%)	1,935 (2.4%)
2013	3 (2.3%)	5,846 (7.3%)
2014	10 (7.6%)	5,572 (6.9%)
2015	6 (4.5%)	13,055 (16%)
2016	32 (24%)	7,444 (9.2%)
2017	14 (11%)	7,297 (9.1%)
2018	13 (9.8%)	8,783 (11%)
2019	10 (7.6%)	9,976 (12%)
2020	2 (1.5%)	8,257 (10%)
2021	13 (9.8%)	6,461 (8.0%)
2022	27 (20%)	5,900 (7.3%)
Dechallenge outcome		
Does not apply	10 (7.6%)	7,753 (9.6%)
Negative dechallenge	0 (0%)	4,102 (5.1%)
Positive dechallenge	59 (45%)	15,754 (20%)
Unknown	63 (48%)	52,917 (66%)
Rechallenge outcome		
Does not apply	10 (7.6%)	19,571 (24%)
Negative rechallenge	0 (0%)	511 (0.6%)
Positive rechallenge	0 (0%)	1,157 (1.4%)
Unknown	122 (92%)	59,287 (74%)
Age group (years)		
< 18	3 (2.3%)	4,770 (5.9%)
18 ~ 45	24 (18%)	19,252 (24%)
45 ~ 64	54 (41%)	20,120 (25%)
65 ~ 75	16 (12%)	7,650 (9.5%)
> = 75	12 (9.1%)	8,373 (10%)
Unknown	23 (17%)	20,361 (25%)
Gender		
Female	105 (80%)	51,097 (63%)
Male	6 (4.5%)	22,815 (28%)
Unknown	21 (16%)	6,614 (8.2%)
Reporter occupation type		
Consumer	9 (6.8%)	33,482 (42%)
Lawyer	0 (0%)	2,313 (2.9%)
Other health-professional	55 (42%)	17,240 (21%)
Pharmacist	5 (3.8%)	5,292 (6.6%)
Physician	62 (47%)	19,448 (24%)
Unknown	1 (0.8%)	2,751 (3.4%)
Region of report		
Africa	0 (0%)	225 (0.3%)
Asia	6 (4.5%)	3,504 (4.4%)
Europe	55 (42%)	37,822 (47%)
North America	52 (39%)	36,481 (45%)
South America	3 (2.3%)	851 (1.1%)
Oceania	12 (9.1%)	613 (0.8%)
Unknown	4 (3.0%)	1,030 (1.3%)
Onset time (days)		
Median (IQR)	20 (20, 41)	13 (0, 151)

### Disproportionality analysis

**Figure 2:**
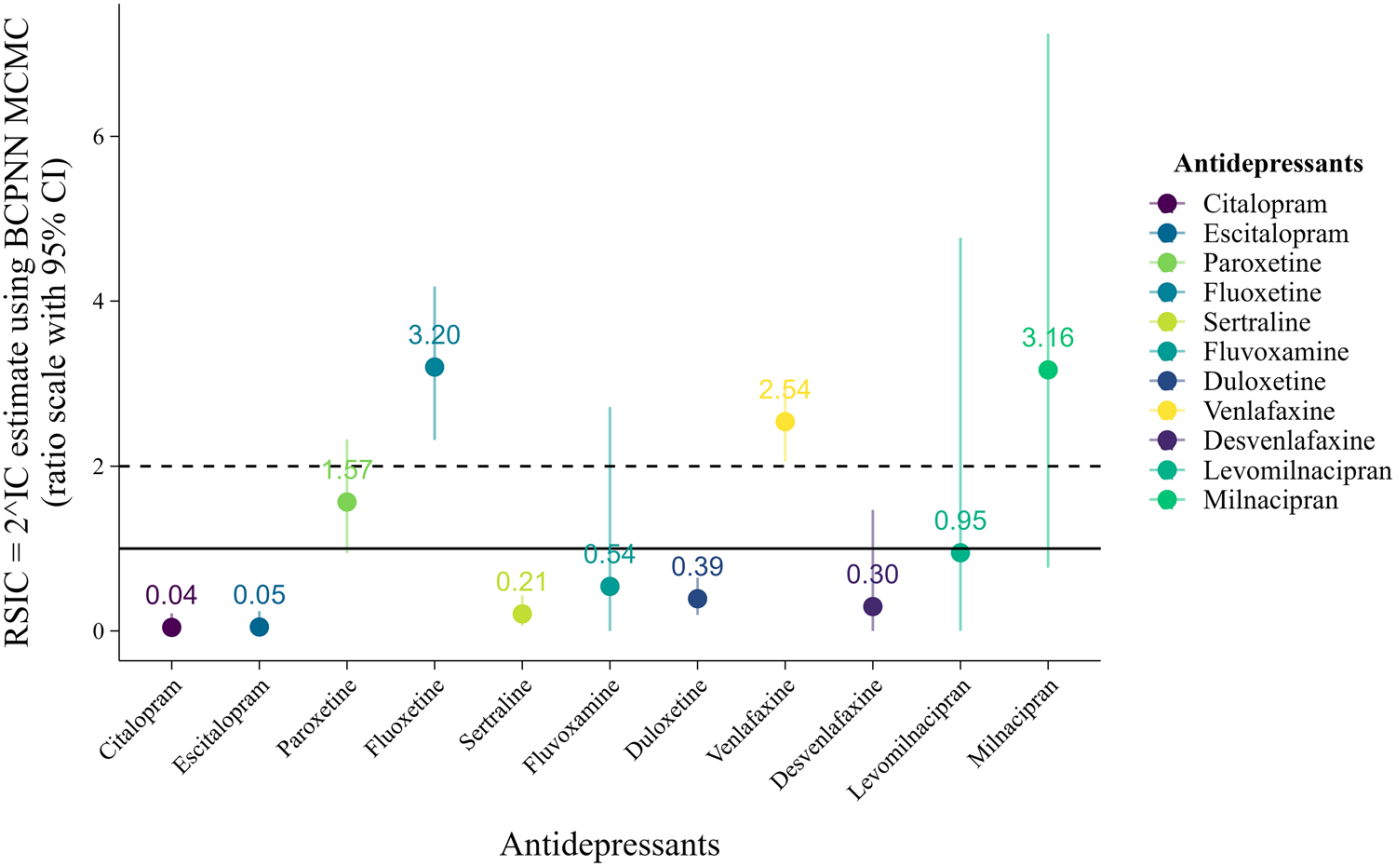
Signal detection estimates and 95% confidence intervals for SC associated with SSRIs and SNRIs.

The results of the disproportionality analysis are summarized in Table S2. Compared with all other antidepressants, reports of SC for venlafaxine and fluoxetine were significantly disproportionate. The RSIC estimates were 2.54 (95% CI 2.06–3.04) for venlafaxine and 3.20 (95% CI 2.31–4.17) for fluoxetine. The disproportionate estimates for the other antidepressants were not significant; none met the signaling threshold (Fig. 2).

**Figure 3:**
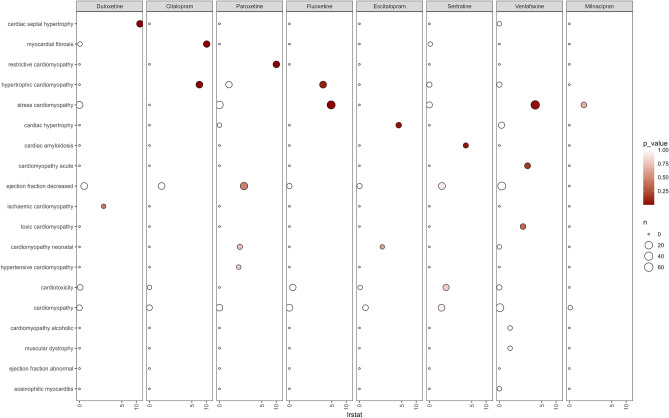
Signal detection estimates for cardiomyopathy (SMQ) associated with SSRIs and SNRIs using likelihood ratio test.

Because drug-induced cardiomyopathy might not only be limited to SC but also included other cardiac symptoms and signs, a likelihood ratio test for cardiomyopathy (SMQ) based on drug–ADR pairs was performed. Compared to all other antidepressants, venlafaxine and fluoxetine showed significant disproportionality in SC, as evidenced by an LRT estimate of 3.55 (p = 0.02) for venlafaxine, and 4.82 (p = 0.008) for fluoxetine (Fig. 3). Our analysis also revealed significant disproportionality between other antidepressants and cardiomyopathy. Duloxetine was linked to cardiac septal hypertrophy, citalopram was associated with both myocardial fibrosis and hypertrophic cardiomyopathy, and paroxetine showed significant disproportionality in restrictive cardiomyopathy. The detailed results are presented in Table S3.

### Concomitant medication

**Figure 4:**
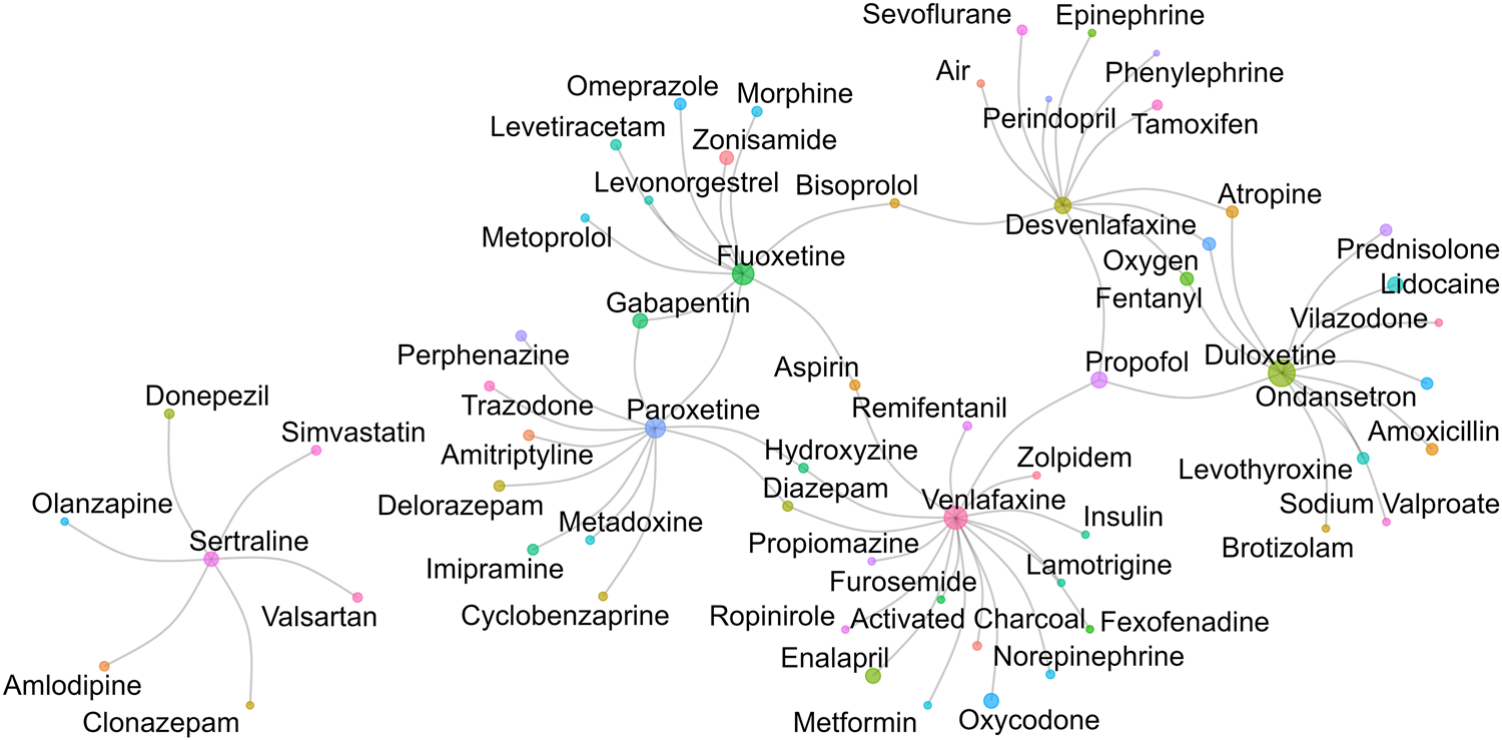
The network diagram of suspected drugs and concomitant drugs. The circles denoted medications, and the lines connecting the circles indicated co-administration relationships between them. The size of the circles represented the frequency of drug use, with larger circles indicating higher usage frequency.

Of the 132 ICSRs, only 29 were reported to be monotherapies; the remaining cases included concomitant medications. The top five concomitant medications for primary and secondary suspected drugs are summarized in Table 2. Among the concomitant medications, nervous system drugs were used most frequently, mainly anesthetics, analgesics, antiepileptics, and psycholeptics. Typical combinations of concomitant drugs included venlafaxine with enalapril or oxycodone, duloxetine with lidocaine or propofol, and fluoxetine with zonisamide or gabapentin (Fig. 4).

**Table 2:** The top 5 concomitant medications with primary and second suspected drugs.

PS/SS drug	Concomitant	Severity*	Frequency
Desvenlafaxine	Tamoxifen	Minor	7
	Sevoflurane	Moderate	7
	Propofol	Moderate	7
	Fentanyl	Moderate	7
	Oxygen	–	5
Venlafaxine	Enalapril	–	31
	Oxycodone	Minor	30
	Propofol	Moderate	5
	Activated charcoal	–	4
	Norepinephrine	Moderate	4
Duloxetine	Lidocaine	Moderate	34
	Propofol	Minor	26
	Atropine	Minor	13
	Ondansetron	Moderate	13
	Prednisolone	–	13
Fluoxetine	Zonisamide	Moderate	23
	Gabapentin	Moderate	19
	Omeprazole	Major	13
	Levetiracetam	Moderate	9
	Morphine	Moderate	8
Paroxetine	Gabapentin	Moderate	10
	Delorazepam	Moderate	10
	Imipramine	Moderate	10
	Perphenazine	Major	9
	Amitriptyline	Moderate	9
Sertraline	Amlodipine	Moderate	6
	Simvastatin	Moderate	6
	Donepezil	Moderate	6
	Clonazepam	Moderate	2
	Olanzapine	Moderate	2

*The severity of the interaction results obtained from the DrugBank database.

### Outcomes

The prognostic outcomes of SC associated with SNRIs and SSRIs are showed in Table 3. Of 132 ICSRs, 180 outcome events were reported. The most frequently reported outcome was initial or prolonged hospitalization (87 cases, 48.33%). Other serious outcomes, including important medical events, were documented in 65 cases (36.11%); life-threatening conditions were recorded in 26 cases (14.44%). Among the outcome events, venlafaxine was reported most frequently (n = 99 cases), followed by fluoxetine (n = 44) and paroxetine (n = 18). Mortalities were reported for the patients who received duloxetine or sertraline.

**Table 3:** The prognostic outcomes of SC associated with SNIRs and SSRIs.

Outcome	Duloxetine	Fluoxetine	Milnacipran	Paroxetine	Sertraline	Venlafaxine
	n = 12	n = 44	n = 3	n = 18	n = 4	n = 99
Death	1 (8.3%)	0 (0%)	0 (0%)	0 (0%)	1 (25%)	0 (0%)
Hospitalization-initial or prolonged	3 (25%)	24 (55%)	2 (67%)	12 (67%)	1 (25%)	45 (45%)
Life-Threatening	4 (33%)	6 (14%)	0 (0%)	0 (0%)	1 (25%)	15 (15%)
Other serious (important medical event)	4 (33%)	14 (32%)	1 (33%)	6 (33%)	1 (25%)	39 (39%)

## Discussion

One hypothesis has suggested that depression results from an imbalance of chemicals in the brain, particularly serotonin, which significantly influences the medical landscape. This perspective has played a significant role in the widespread prescription of antidepressants worldwide^12^. Among antidepressants, SSRIs and SNRIs are the most prevalent because they have fewer side effects than earlier antidepressants. Although clinical evidence suggests that SSRIs and SNRIs are generally safe for patients, recent case reports have shown that antidepressants may induce SC. In a real-world clinical scenario, observing SC induced by these types of antidepressants is not straightforward. Therefore, a disproportionality analysis was performed based on the FAERS database, and the association between SC and using SSRIs or SNRIs was explored.

This study identified 132 ICSRs related to SSRIs and SNRIs involving SC. More cases were associated with SNRIs than SSRIs; venlafaxine (n = 63, 48%) and fluoxetine (n = 35, 27%) were particularly prevalent. The incidence of SC showed a significant difference between the genders; susceptibility to SC was greater in female patients than in male patients. Female patients accounted for 80% of the total, generally consistent with the cohort observations^13^. Mental disorders and menopausal estrogen deficiency are important pathogenic factors in SC^14^. Females are more prone to depression than males because of variations in estrogen levels across lifespans^15^.

Our results confirmed that SC associated with SSRIs or SNRIs was more prevalent in young and middle-aged adults (18% aged 18–45 years and 41% aged 45–65 years). However, studies have suggested that SC predominates in menopausal women, with a mean age of 65 years or even older^13,14,16^. The change in the risk of affective disorders in menopausal women, contrasted with the slower and less-pronounced variations in psychosocial factors, favors the hormonal argument. However, the risk of affective disorders is lower in both sexes after the age at which menopause occurs in women, decreasing the appeal of the hormonal interpretation^17^. An alternative explanation is that sympathetic stimulation and alterations in the autonomic nervous system are involved in the pathogenesis of SC^18^; age-related changes may occur in sympathetic stimulation or myocardial susceptibility to catecholamine excess^19^. A cohort study that included 2098 patients with Takotsubo syndrome identified age-specific clinical features. Younger patients at high risk for acute neurological or psychiatric disorders were more susceptible to sympathetic stimulation than middle-aged and older patients with Takotsubo syndrome^20^. However, this finding requires validation in a large cohort study.

A notable variation is evident in reports of antidepressant use associated with cardiomyopathic across different geographic regions, particularly in Europe and North America. Europeans prefer venlafaxine and paroxetine; North Americans seem likely to use both fluoxetine and sertraline. Our results are generally consistent with multiple studies^21–24^. Such geographical discrepancies were confined to differences between countries and were pronounced within individual countries^25,26^. Regional variations in antidepressant consumption might result from the interplay of individual health care-seeking behaviors, local culture and customs, prescribing behaviors, and health care organizations. Despite the differing preferences for antidepressant use observed between the two regions, the proportion of SC reports was similar in Europe (78.2%) and North America (82.7%). The majority of reports in both regions involved postmenopausal women, suggesting that the triggers for SC may extend beyond drug factors; estrogen deficiency potentially plays a crucial role in postmenopausal women.

Our analysis identified a potential association between using fluoxetine and venlafaxine and SC. Disproportionality analysis revealed a significant risk for these two antidepressants. Although other SSRIs or SNRIs may contribute to myocardial injury^27^, our findings do not indicate an association with SC events. The pathophysiological mechanisms underlying SC remain elusive, multiple hypotheses are being investigated. These hypotheses include excessive sympathetic nervous system activation, catecholamine toxicity, myocardial stunning, estrogen deficiency, and genetic predisposition linked to coronary artery structural abnormalities. Cardiac toxicity resulting from sympathetic overstimulation by catecholamines is one of the most widely accepted mechanisms of action^28^. Venlafaxine, an SNRI, suppresses catecholamines reuptake by presynaptic neurons, increasing the synaptic concentrations of norepinephrine and serotonin, potentially causing iatrogenic catecholamine excess and SC. Our findings revealed a significant risk for SC associated with fluoxetine, a potent selective serotonin reuptake inhibitor. While fluoxetine was thought to have a negligible effects on norepinephrine, Bymaster et al. reported that it increases the extracellular levels of norepinephrine and dopamine in the prefrontal cortex, suggesting a potential mechanism for its role in SC onset^29^.

Our findings showed a significant association between ventricular septal hypertrophy and duloxetine, raising concerns about the etiology of acute left ventricular ballooning. The prevailing hypothesis suggests ballooning is caused by direct catecholamine toxicity on cardiomyocytes or by microvascular ischemia. More recently, another possibility has emerged: in individuals with underling hypertrophic cardiomyopathy, left ventricular ballooning might be triggered by a sudden onset of latent left ventricular outflow tract obstruction. When it becomes severe and unrelenting, severe afterload mismatch and acute supply demand ischemia result in ballooning^30^. The clinical features of patients with ballooning due to these two distinct causes can overlap, potentially leading to underdiagnosis and misclassification. Both syndromes might by triggered by an excess of catecholamine from stress. Specifically, SC is caused by direct toxicity or vasospasm, while obstructive hypertrophic cardiomyopathy with left ventricular ballooning is induced by severe pressure gradients. A through assessment of outflow tract obstruction and echocardiographic assessment are essential for differential diagnosis in clinical practice. The observed association between ventricular septal hypertrophy and duloxetine does not necessarily imply causation and warrants further validation.

The co-administration of multiple medications can significantly influence serotonin levels. The simultaneous use of ≥ 2 serotonergic drugs, even at therapeutic doses, can cause moderate-to-severe serotonin surges, resulting in serious adverse consequences and mortality^31^. Abnormally elevated levels of catecholamines and/or serotonin, often because of drug interactions or an overdose of SSRIs/SNRIs, are suspected contributors to SC^5,32–34^. These findings raise significant safety concerns regarding these medications^2,14^. This study found that a significant proportion (78.03%) of patients with SC treated with antidepressants had used other medications concurrently. The five most frequently co-administered medications were propofol, lithium, oxycodone, gabapentin, and zonisamide. These medications are either known or suspected to possess serotonergic properties^35–38^, raising concerns about their potential contribution to the serotonin syndrome. Serotonin syndrome can manifest as hyperadrenergic and serotonergic states, both of these are potential triggers of SC^39^. The relationship between serotonin syndrome and SC has not been clarified. Mehta et al. suggested that a serotonin syndrome-induced hyperadrenergic state can cause physiological stress, triggering SC^39,40^. Alternatively, excessive serotonin levels may directly overstimulate serotonin receptors in the heart, contributing to SC development^40^. Thus, the severity of the outcomes may especially increase when serotonergic drugs are combined with SSRIs or SNRIs^41,42^. Therefore, SC should be considered a significant concern when SSRIs or SNRIs are administered with serotonin reuptake inhibitors or drugs that clinically influence the activity of cytochrome P450 enzymes. Specialized physicians or pharmacists should be carefully evaluate the medication regimens.

SC is considered reversible in clinical practice; most patients recover with a favorable prognosis after symptomatic treatment. However, our results indicated that initial or prolonged hospitalization and other serious events were common prognostic outcomes of SC induced by SSRIs or SNRIs. Studies suggest that the mortality rate or incidence of adverse cardiovascular events in SC is comparable to coronary heart disease, challenging the widely held belief that SC is benign. The incidence rates of mortality, heart failure-related hospitalization, and SC recurrence in survivors among patients with SC are 6.9, 0.9, and 1.1 events per 100 person-years^43^. Survivors of SC have greater associated mortality rates (adjusted hazard ratio 2.05, 95% CI, 1.62–2.60) and heart failure-related hospitalization (adjusted hazard ratio 4.24, 95% CI 1.88–9.53) than the background populations^43^. Therefore, initiating diagnosis as early as possible is imperative to prevent misdiagnosis. Appropriate diagnostic and therapeutic strategies should be formulated based on a patient’s condition to improve prognosis.

This study has some limitations. First, our study was based on a spontaneously reported adverse-events database; including reporting bias, missing items, and entry errors, could introduce bias and confounding the outcomes. Second, several statistical results might be biased because of missing records; therefore, the results must be interpreted with cautiously. Although stratification by age and gender was employed to minimize these biases, the pharmacovigilance analysis performed using the FAERS database could not fully control for confounders that may affect the causal relationship between drugs and adverse events. This study identified a potential association between fluoxetine and SC. However, the precise triggering mechanisms remain unclear. Undiscovered drug mechanisms, drug interactions, individual genetic susceptibilities, and pre-existing conditions may all contribute, necessitating further research. Moreover, the potential interplay between serotonin syndrome and SC cannot be elucidated through pharmacovigilance analysis alone; extensive foundational and clinical research is required for validation. Third, the SC risk signals reported in this study indicated only a statistical association between drugs and adverse events; a causal relationship needs to be verified. Finally, the database included only reported adverse events and did not fully correspond to the actual incidence of adverse events in the population. Thus, the prevalence of SC in the population needs further study.

## Conclusion

Based on the analysis of the FAERS database, this study provides comprehensive insights into the characteristics of SC associated with SSRIs or SNRIs, revealing potential differences between SSRIs and SNRIs in the risk of including SC, especially in females. Our results suggest that venlafaxine and fluoxetine are significant risk factors; however, this finding needs to be confirmed via large epidemiological studies.

### Supplementary Information

Supplementary Tables.

## Data Availability

The raw data used and/or analyzed during the current study are available from the FAERS quarterly data extract files website (https://fis.fda.gov/extensions/FPD-QDE-FAERS/FPD-QDE-FAERS.html).
