# Supplementary material for: Risk of stress cardiomyopathy associated with selective serotonin reuptake inhibitors and serotonin and norepinephrine reuptake inhibitors: a real-world pharmacovigilance analysis
Source: Sci Rep. 2024 Jul 2;14:15167. doi: 10.1038/s41598-024-66155-1 (PMC11220088; doi:10.1038/s41598-024-66155-1)
Supplement: Supplementary file 1 — Supplementary Tables. [file 41598_2024_66155_MOESM1_ESM.docx]

**Table S1.** Preferred terms of cardiomyopathy (SMQ)

| Preferred Terms | Code | Level | Scope |
| --- | --- | --- | --- |
| Chagas' cardiomyopathy | 10080484 | PT | Narrow |
| HIV cardiomyopathy | 10069658 | PT | Narrow |
| Toxic cardiomyopathy | 10083657 | PT | Narrow |
| Metabolic cardiomyopathy | 10070909 | PT | Narrow |
| Congestive cardiomyopathy | 10056370 | PT | Narrow |
| Kearns-Sayre syndrome | 10048804 | PT | Narrow |
| Right ventricular ejection fraction decreased | 10075337 | PT | Narrow |
| Eosinophilic myocarditis | 10014961 | PT | Narrow |
| Peripartum cardiomyopathy | 10049430 | PT | Narrow |
| Ejection fraction abnormal | 10014331 | PT | Narrow |
| Ejection fraction decreased | 10050528 | PT | Narrow |
| Giant cell myocarditis | 10083635 | PT | Narrow |
| Stress cardiomyopathy | 10066286 | PT | Narrow |
| Tachycardia induced cardiomyopathy | 10074269 | PT | Narrow |
| Biopsy heart abnormal | 10004780 | PT | Narrow |
| Myocardial haemorrhage | 10048849 | PT | Narrow |
| Cardiomyopathy | 10007636 | PT | Narrow |
| Myocardial fibrosis | 10028594 | PT | Narrow |
| Myocardial calcification | 10054122 | PT | Narrow |
| Cardiotoxicity | 10048610 | PT | Narrow |
| Cardiac amyloidosis | 10007509 | PT | Narrow |
| Cardiac sarcoidosis | 10007604 | PT | Narrow |
| Cardiac hypertrophy | 10007572 | PT | Narrow |
| Cardiac iron overload | 10080569 | PT | Narrow |
| Cardiac septal hypertrophy | 10057576 | PT | Narrow |
| Cardiomyopathy acute | 10048377 | PT | Narrow |
| Cardiomyopathy neonatal | 10050111 | PT | Narrow |
| Thyrotoxic cardiomyopathy | 10075043 | PT | Narrow |
| Viral cardiomyopathy | 10068767 | PT | Narrow |
| Diabetic cardiomyopathy | 10012647 | PT | Narrow |
| Mitochondrial cardiomyopathy | 10084364 | PT | Narrow |
| Ischaemic cardiomyopathy | 10048858 | PT | Narrow |
| Muscular dystrophy | 10028356 | PT | Narrow |
| Hypertrophic cardiomyopathy | 10020871 | PT | Narrow |
| Obesity cardiomyopathy | 10081007 | PT | Narrow |
| Pulmonary arterial wedge pressure increased | 10037329 | PT | Narrow |
| Septic cardiomyopathy | 10087221 | PT | Narrow |
| Ventricular septal defect acquired | 10047299 | PT | Narrow |
| Atrial septal defect acquired | 10003665 | PT | Narrow |
| Pacing induced cardiomyopathy | 10086997 | PT | Narrow |
| Cardiomyopathy alcoholic | 10007637 | PT | Narrow |
| Restrictive cardiomyopathy | 10038748 | PT | Narrow |
| Non-obstructive cardiomyopathy | 10049813 | PT | Narrow |
| Hypertensive cardiomyopathy | 10058222 | PT | Narrow |

**Table S2.** The results of disproportionality analysis of SSRIs/SNRIs associated stress cardiomyopathy.

| **Drug** | **a** | **b** | **c** | **d** | **ROR** | **PRR** | **EBGM** | **RSIC = 2**IC |
| --- | --- | --- | --- | --- | --- | --- | --- | --- |
| Citalopram | 0 | 37377 | 162 | 499149 | - | - | - | 0.04 (0.00, 0.21) |
| Escitalopram | 0 | 33071 | 162 | 503455 | - | - | - | 0.05 (0.00, 0.24) |
| Sertraline | 4 | 70379 | 158 | 466147 | 0.17 | 0.15 | 0.2 | 0.21 (0.06, 0.43) |
| Fluoxetine | 35 | 35104 | 127 | 501422 | 3.08 | 3.65 | 2.89 | 3.20 (2.31, 4.17)* |
| Paroxetine | 17 | 35366 | 145 | 501160 | 1.94 | 2.05 | 1.65 | 1.57 (0.95, 2.32) |
| Venlafaxine | 63 | 81283 | 99 | 455243 | 2.58 | 3.58 | 2.46 | 2.54 (2.06, 3.04)* |
| Desvenlafaxine | 0 | 3951 | 162 | 532575 | - | - | - | 0.30 (0.00, 1.47) |
| Duloxetine | 10 | 87271 | 152 | 449255 | 0.49 | 0.46 | 0.55 | 0.39 (0.19, 0.65) |
| Levomilnacipran | 0 | 89 | 162 | 536437 | - | - | - | 0.95 (0.00, 4.77) |
| Milnacipran | 3 | 2007 | 159 | 534519 | 3.98 | 4.04 | 1.84 | 3.16 (0.77, 7.25) |
| Fluvoxamine | 0 | 1408 | 162 | 535118 | - | - | - | 0.54 (0.00, 2.71) |

Note: a: Targeted antidepressants with stress cardiomyopathy. b: all antidepressants. c: Non-antidepressants with stress cardiomyopathy. d: Non-antidepressants. ROR: Reporting Odds Ratio. PRR: Proportional Reporting Ratio. EBGM: Empirical Bayes Geometric Mean. RSIC: Ratio Scale Information Component. *: denoted as potential signal. -: Not applicable.

**Table S3.** Signal detection estimates for cardiomyopathy (SMQ) associated with SSRIs and SNRIs using likelihood ratio test.

| **AE** | **Drug** | **n** | **LRT** | **p value** |
| --- | --- | --- | --- | --- |
| cardiac septal hypertrophy | Duloxetine | 8 | 11.850 | 0.000 |
| myocardial fibrosis | Citalopram | 7 | 10.201 | 0.000 |
| restrictive cardiomyopathy | Paroxetine | 8 | 10.062 | 0.000 |
| hypertrophic cardiomyopathy | Citalopram | 9 | 7.238 | 0.003 |
| stress cardiomyopathy | Fluoxetine | 35 | 4.820 | 0.008 |
| cardiac hypertrophy | Escitalopram | 3 | 4.261 | 0.029 |
| stress cardiomyopathy | Venlafaxine | 63 | 3.548 | 0.023 |
